# Circulating angiotensin-converting enzyme 2 concentration is associated with acute kidney injury and mortality in sepsis

**DOI:** 10.1371/journal.pone.0330668

**Published:** 2025-08-29

**Authors:** Ching-En Chen, Ruey-Hsing Chou, Jiun-Yu Guo, Ya-Wen Lu, Chun-Chin Chang, Cheng-Hsueh Wu, Po-Hsun Huang

**Affiliations:** 1 Division of Plastic and Reconstructive Surgery, Department of Surgery, Taipei Veterans General Hospital, Taipei, Taiwan; 2 Institute of Clinical Medicine, National Yang-Ming Chiao-Tung University, Taipei, Taiwan; 3 Cardiovascular Research Center, National Yang-Ming Chiao-Tung University, Taipei, Taiwan; 4 Division of Cardiology, Department of Medicine, Taipei Veterans General Hospital, Taipei, Taiwan; 5 Department of Critical Care Medicine, Taipei Veterans General Hospital, Taipei, Taiwan; 6 Division of Cardiology, Department of Medicine, New Taipei City Hospital, New Taipei City, Taiwan; 7 School of Medicine, College of Medicine, National Yang Ming Chiao Tung University, Taipei, Taiwan; Universitas Muhammadiyah Aceh, INDONESIA

## Abstract

**Background:**

Beyond the well-established classical renin-angiotensin system (RAS), emerging evidence highlights the critical role of the non-classical RAS, specifically the Angiotensin (1–7)/ACE2/Mas axis. As the key enzyme converting Angiotensin II into Angiotensin (1–7), angiotensin-converting enzyme 2 (ACE2) exerts cardioprotective and anti-inflammatory effects, showing potential therapeutic value in critical care. This study investigates the association between circulating ACE2 levels and clinical outcomes in sepsis, offering insights into its role and potential for predicting sepsis outcomes.

**Materials and methods:**

In this single-center study, we investigated associations between the circulating ACE2 concentration and outcomes in septic patients admitted to a medical intensive care unit (ICU) between 01/05/2018 and 31/01/2021. Sepsis was defined as infection accompanied by a ≥2-point Sequential Organ Failure Assessment (SOFA) score increase. Patients were categorized into low (<2.5 ng/mL) and high (≥2.5 ng/mL) ACE2 groups based on serum concentrations within 24 hours of ICU admission. Outcomes comprised acute kidney injury (AKI), ICU mortality, and 90-day mortality.

**Results:**

In total, 414 patients (mean age 68.5 years, 64.7% male) were included in the study. Elevated ACE2 levels correlated positively with SOFA score and total bilirubin and lactate concentrations, and negatively with the hemoglobin concentration. Relative to the low ACE2 group, the high ACE2 group was at increased risk of sepsis-associated AKI development within 48 hours after ICU admission (81.6% vs. 69.6%, *p* = 0.006), AKI requiring renal replacement therapy (21.3% vs. 11.1%, *p* = 0.007), ICU mortality (31.9% vs. 17.5%, *p* = 0.001), and 90-day mortality (51.7% vs. 39.6%, *p* = 0.018). Kaplan-Meier survival curves demonstrated significantly reduced survival in individuals with high ACE2 concentrations (*p* = 0.009). Univariate analysis revealed significant associations of high ACE2 concentrations with ICU mortality and AKI development within 48 hours after ICU admission. In a multivariate analysis adjusted for relevant variables, ACE2 elevation remained an independent predictor of ICU mortality (adjusted odds ratio 2.15, 95% confidence interval 1.04–4.41, *p* = 0.038).

**Conclusion:**

Elevated circulating ACE2 concentrations comprised an independent predictor of ICU mortality, highlighting intricate RAS dynamics in critical illnesses. Low ACE2 concentrations were associated with greater survival, suggesting their potential as an early prognostic indicator.

## Introduction

The intricate regulation of blood pressure and electrolyte balance is governed by the classic renin-angiotensin system (RAS), and specifically by the well-established angiotensin II (Ang II)/angiotensin-converting enzyme (ACE)/angiotensin II receptor type 1 (AT1) axis, with physiological responses mediated through the octapeptide Ang II [1,2]. However, recent research has brought attention to an alternative RAS pathway, often referred to as the non-classic cascade: the Ang-(1–7)/ACE2/Mas axis [1,3]. This counterregulatory mechanism operates independently of the classic axis, contributing to RAS regulation with distinct implications [4,5]. Deeper understanding of the physiology of the entire RAS, particularly the role of non-classic cascade, has stimulated the development of novel prognostic and therapeutic strategies [1].

Angiotensin-converting enzyme 2 (ACE2) is widely expressed in organs such as the kidneys, lungs, brain, heart, and testes, plays a pivotal role in catalyzing the conversion of Ang II to Ang-(1–7) and Ang I to Ang-(1–9) [1,4]. Notably, the conversion of Ang II to Ang-(1–7) by ACE2 establishes the non-classical RAS pathway, which has been implicated in protective mechanisms against various diseases [1]. For example, the cardioprotective and anti-inflammatory activities of Ang-(1–7) and ACE2 have demonstrated efficacy in experimental models of myocardial infarction and heart failure [6,7]. Moreover, ACE2 has been recognized as a target for the prevention of pancreatic beta-cell dysfunction and improvement of fasting glycemia in diabetic mice [8]. Its critical role in renal disease is also evident, as studies have linked ACE2 expression to the onset and progression of renal dysfunction in various diseases [9].

Given the antagonism between the classic and non-classic RAS cascades, we propose that the circulating concentration of the key enzyme ACE2 could serve as a prognostic biomarker, particularly relevant in intensive care setting. To date, there is currently a lack of studies exploring its role in predicting outcomes in sepsis patients, underscoring the novelty of our investigation. Hence, we conducted this study to elucidate the nuanced relationship between circulating ACE2 concentrations at the onset of sepsis and subsequent clinical outcomes, potentially offering valuable insights into the evolving landscape of critical care.

## Materials and methods

### Study population and data collection

We conducted a post-hoc analysis of retrospective cohort data from a single-center biobank study involving patients admitted to our intensive care unit (ICU) between 01/05/2018 and 31/01/2021. Out of 463 patients screened upon ICU admission, 49 were excluded due to either a lack of confirmed sepsis or a history of regular renal replacement therapy, resulting in the inclusion of 414 cases. Data extracted from electronic medical records included subjects’ age, sex, Sequential Organ Failure Assessment (SOFA) score, vasopressor/inotrope usage, fluid resuscitation within 24 hours, invasive mechanical ventilation, and mean arterial pressure (MAP), recorded during the initial assessment at ICU admission, either before or during the first fluid resuscitation. Common pre-existing comorbidities (hypertension, diabetes mellitus, heart failure, chronic kidney disease, liver cirrhosis, and active malignancy) were identified. Patients with usage of ACEi (angiotensin converting enzyme inhibitors) or ARB (angiotensin II receptor blockers) was defined as those who used the medicines for more than 1 month before ICU admission. Infections were categorized according to their source as upper or lower respiratory tract infections (RTIs), urinary tract infections (UTIs), intra-abdominal infections (IAIs), and primary bacteremia (originating directly from the bloodstream). RTIs were diagnosed by clinical presentations such as the common cold, rhinosinusitis, tracheitis, bronchiolitis, and pneumonia, and confirmed by radiographic findings and/or bacterial culture. UTIs were diagnosed by combined clinical evaluation (urinalysis findings of pyuria, hematuria, bacteriuria, or other indicators of infection) and positive urine culture results. Data on laboratory parameters, including the peripheral blood white blood cell count (WBC) and hemoglobin (Hb), serum creatinine (Cr) and total bilirubin (TBIL) concentrations, were collected. Missing laboratory data were replaced with the mean from all study subjects. The numbers of subjects with missing data are reported in S1 Table.

Sepsis was defined according to the 2016 guidelines of the Surviving Sepsis Campaign and the criteria of the Third International Consensus Definitions for Sepsis and Septic Shock, with the diagnoses of sepsis and septic shock confirmed by organ dysfunction (≥2-point increase in the SOFA score) and the use of vasopressor or inotrope, respectively in the first 24 hours after ICU transference [10,11]. The SOFA score evaluates organ dysfunction by grading abnormalities across six components: respiratory function (PaO2/FiO2), coagulation (platelet count), liver function (bilirubin), cardiovascular status (hypotension or inotropic support), central nervous system (Glasgow Coma Scale), and renal function (creatinine or urine output).[11] The institutional review board of Taipei Veterans General Hospital approved the study protocol (Num. 2018-02-009 AC). All participants provided written informed consent, and this study adhered to the principles outlined in the Declaration of Helsinki.

### Serum ACE2 measurement

Peripheral blood samples collected from patients within the first 24 hours of ICU admission were retrieved from the biobank. They were allowed to stand for 1 hour and then centrifuged at 3,000 rpm and 4°C for 10 minutes for supernatant (serum) collection. Serum aliquots (250 µL) were stored at –80°C until serum biomarker analysis.

**Fig 1:**
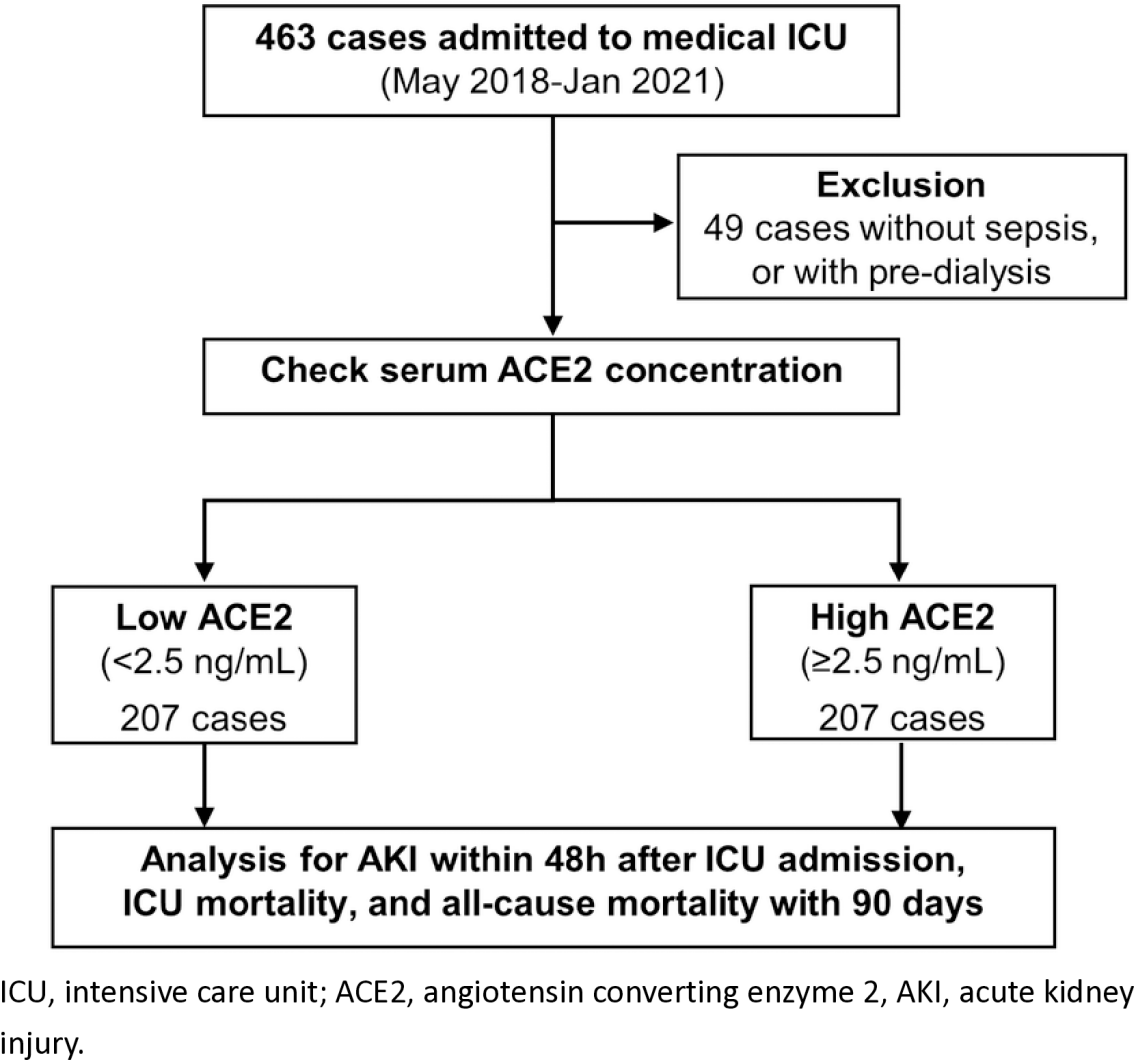
Flowchart of patient enrollment and classification.

Serum ACE2 activity was determined using a commercial enzyme-linked immunosorbent assay (R&D Systems, Inc., Minneapolis, MN, USA). The sensitivity was 0.23 ng/mL and the assay range was 0.3–20 ng/mL. Intra- and inter-assay coefficients of variability were <4.99% and <5.43%, respectively. Enrolled patients were divided into two equally sized groups based on the median serum ACE2 concentration (Fig 1). Subjects with serum ACE2 concentrations < 2.5 ng/mL comprised the low ACE2 group (*n* = 207) and those with concentrations ≥ 2.5 ng/mL constituted the high ACE2 group (*n* = 207).

### Patient follow up and clinical outcomes

The study outcomes were sepsis-associated acute kidney injury (AKI), lengths of ICU and hospital stays, mortality during ICU stay, and 90-day mortality. Serum Cr concentrations were measured at the time of ICU admission and daily throughout patients’ ICU stays. AKI was confirmed according to the Kidney Disease: Improving Global Outcomes guideline [12] within 48 hours after ICU admission, via a ≥ 0.3 mg/dL increase in the serum Cr concentration within 48 hours; a ≥ 1.5-fold increase in the serum Cr concentration from baseline, known or presumed to have occurred within the previous 7 days; or a urine volume < 0.5 mL/kg/hour for 6 hours. All subjects were followed for mortality during their ICU stays and for at least 90 days after ICU admission.

### Statistical analysis

Statistical significance was defined as *p* < 0.05. Categorical variables were represented as proportions and analyzed using Fisher’s exact test. Continuous variables were expressed as medians with interquartile ranges and evaluated using the Mann–Whitney *U* test. Survival analysis was conducted using Kaplan–Meier curves and the log-rank test to estimate the mortality rates. The sample size calculation was based on the assumption of two independent study groups, with the primary outcome being short-term mortality. As there was currently no available data for septic patients stratified by ACE2 levels, we calculated sample size by using data from critically ill COVID-19 patients [13]. The reported 30-day mortality was 37.3% in critically ill patients with low circulating ACE2 levels, and 64.7% in those with high ACE2 levels. Using the two-sample Mann–Whitney *U* test, a total of 136 subjects were required to achieve a power of 0.90 with a target significance of 0.05. Considering the prevalence of sepsis was 44% in our previous work [14], we needed to screen at least 309 ICU-admitted patients to complete this study. Ultimately, our study included 414 participants, which exceeds the required sample size and ensures sufficient power to detect significant effects.

Risk factors associated with ICU mortality were assessed using forward stepwise logistic regression, with odds ratios (ORs) and 95% confidence intervals (CIs) reported. Variables with *p* values < 0.05 in univariate regression analyses were included in the multivariate regression model. Subgroup analyses were performed by stratifying patients according to age and sepsis severity, as defined by the presence or absence of septic shock. Sensitivity analyses were also conducted using different ACE2 classifications. In addition to divide the cohort into two equal groups by the median ACE2 value, we divided the cohort into ACE2 tertiles in the sensitivity analyses. We hypothesized that relationships between the ACE2 concentration and clinical outcomes, including ICU mortality and AKI within 48 hours after ICU admission, would be non-linear. To explore this hypothesis, we conducted restricted cubic spline analysis [15], with five knots placed at the 2nd, 25th, 50th, 75th, and 98th percentiles of the serum ACE2 concentration. A cubic spline curve was generated using the RStudio interface for R (version 3.6.3; R Core Team, Vienna, Austria). These analyses were performed using SPSS software (version 19.0; IBM Corporation, Armonk, NY, USA).

## Results

### Cohort characteristics

The median age of the study cohort was 68.5 years, and 268 (64.7%) of the 414 subjects were male. Most (79.2%) infections were RTIs, followed by bloodstream infections (27.1%), IAIs (15.5%), and UTIs (14.5%). The low and high ACE2 groups did not differ in terms of age, sex distribution, mean arterial pressure, vasopressor/inotrope usage, fluid resuscitation within 24 hours, invasive mechanical ventilation, or prevalence of underlying diseases such as diabetes mellitus, heart failure, chronic kidney disease, liver cirrhosis, and active malignancy (Table 1). Additionally, no significant difference in laboratory parameters, including the WBC and serum Cr concentration within the first 24 hours after ICU admission, was observed between groups. Significant differences were observed between the high and low ACE2 groups in the SOFA score (9.5 vs. 9.5, **p* *< 0.001), proportion of bloodstream infections (31.9% vs. 22.2%, **p* *= 0.035), underlying hypertension (44.0% vs. 56.0%, **p* *= 0.018), Hb concentration (8.6 vs. 9.2 mg/dL, **p* *= 0.002), TBIL concentration (1.2 vs. 0.7 mg/dL, **p* *< 0.001), and lactate concentration (16.2 vs. 12.6 mg/dL, **p* *= 0.015; Table 1).

**Table 1:** Demographic characteristic of critically-ill patients grouped by serum angiotensin converting enzyme 2 (ACE2) concentrations. Continuous variables are expressed as medians (interquartile ranges); and categorical variables are expressed as numbers (percentage).

	Total population N = 414	Low serum ACE2 (<2.50 ng/mL) N = 207	High serum ACE2 (≥2.50 ng/mL) N = 207	*P* value
Age (years)	68.5 (59.0-79.0)	67.0 (59.0-78.0)	70.0 (59.0-80.0)	0.600
Male gender	268 (64.7)	143 (69.1)	125 (60.4)	0.080
SOFA scores	9.5 (8.0-11.0)	9.5 (7.0-10.0)	9.5 (9.0-12.0)	<0.001
Main arterial pressure (mmHg)	55.7 (48.5-65.0)	55.0 (47.3-64.3)	55.7 (49.3-66.0)	0.094
Vasopressor/inotrope usage	165 (39.9)	81 (39.1)	84 (40.6)	0.841
Fluid resuscitation within 24h (L)	1.8 (1.0-3.0)	1.9 (1.0-2.7)	1.7 (0.9-3.3)	0.580
Invasive mechanical ventilation	370 (89.4)	187 (90.3)	183 (88.4)	0.633
**Sources of Infection**				
Respiratory tract infection	328 (79.2)	167 (80.7)	161 (77.8)	0.545
Urinary tract infection	60 (14.5)	27 (13.0)	33 (15.9)	0.485
Intra-abdominal infection	64 (15.5)	32 (15.5)	32 (15.5)	1.000
Bloodstream infection	112 (27.1)	46 (22.2)	66 (31.9)	0.035
**Co-morbidities**				
Hypertension	207 (50.0)	116 (56.0)	91 (44.0)	0.018
Diabetic mellitus	145 (35.1)	82 (39.6)	63 (30.4)	0.063
Heart failure	57 (13.8)	31 (15.0)	26 (12.6)	0.569
Chronic kidney disease	134 (32.4)	72 (34.8)	62 (30.0)	0.344
Cirrhosis	28 (6.8)	9 (4.3)	19 (9.2)	0.077
Malignancy	143 (34.5)	67 (32.4)	76 (36.7)	0.408
Usage of ACEi/ ARB	97 (23.4)	49 (23.7)	48 (23.2)	1.000
**Lab data**				
White blood cells (K)	9.1 (5.3-14.2)	9.4 (5.3-14.1)	8.7 (5.3-14.3)	0.904
Hemoglobin (mg/dL)	8.9 (7.9-10.1)	9.2 (8.1-10.7)	8.6 (7.6-9.9)	0.002
Creatinine (mg/dL)	1.9 (1.1-2.8)	1.8 (1.0-2.6)	2.0 (1.1-2.9)	0.421
Total bilirubin (md/dL)	0.9 (0.5-2.2)	0.7 (0.5-1.6)	1.2 (0.6-2.9)	<0.001
Lactate (mg/dL)	14.4 (8.1-18.9)	12.6 (8.1-18.9)	16.2 (9.0-21.6)	0.015
ACE2 (ng/mL)	2.5 (1.1-6.6)	1.1 (0.7-1.7)	6.5 (3.9-16.5)	<0.001

SOFA, Sequential Organ Failure Assessment; ACEi, Angiotensin converting enzyme inhibitors; ARB, angiotensin II receptor blockers.

### Correlations of clinical variables with the ACE2 concentration

The serum ACE2 concentration correlated positively with the SOFA score (*r* = 0.183, **p* *< 0.001), TBIL concentration (*r* = 0.274, **p* *< 0.001), and lactate concentration (*r* = 0.131, **p* *= 0.008), and negatively with the Hb concentration (**r* *= –0.180, **p* *< 0.001; Table 2). It did not correlate significantly with the patient age, mean arterial pressure, fluid resuscitation within 24 hours, WBC, or Cr concentration.

**Table 2:** Correlation coefficients between circulating angiotensin converting enzyme 2 (ACE2) levels and various clinical variables.

	ACE2	
Variable	**r**	***p value***
Age (years)	0.078	0.113
SOFA scores	0.183	<0.001
Main arterial pressure (mmHg)	0.028	0.567
Fluid resuscitation within 24h (L)	0.033	0.499
White blood cells (K)	−0.010	0.832
Hemoglobin (mg/dL)	−0.180	<0.001
Creatinine (mg/dL)	0.028	0.568
Total bilirubin (mg/dL)	0.274	<0.001
Lactate (mg/dL)	0.131	0.008

SOFA, Sequential Organ Failure Assessment.

### Clinical outcomes differed between the low and high ACE2 groups

**Fig 2:**
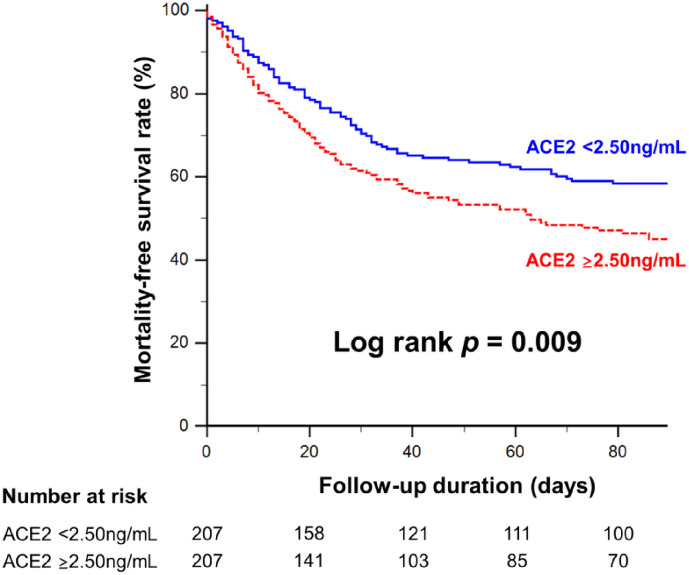
Kaplan–Meier curves of freedom from mortality in critically-ill patients grouped by serum angiotensin converting enzyme 2 (ACE2) concentrations.

Relative to the low ACE2 group, the high ACE2 group had greater rates of AKI within the first 48 hours of ICU admission (81.6% vs. 69.6%, **p* *= 0.006), consequent renal replacement therapy (21.3% vs. 11.1%, **p* *= 0.007), ICU mortality (31.9% vs. 17.5%, **p* *= 0.001), and 90-day mortality (51.7% vs. 39.6%, **p* *= 0.018). No significant difference was observed between groups in the length of ICU (9.0 and 8.0 days, respectively) or hospital (26.0 and 22.5 days, respectively) stays (Table 3). Kaplan-Meier curves confirmed that reduced 90-day survival was associated with high serum ACE2 concentrations relative to low concentrations (log-rank *p* = 0.009; Fig 2).

**Table 3:** Clinical outcomes of critically ill patients grouped by serum angiotensin converting enzyme 2 (ACE2) concentrations.

	Total population N = 414	Low serum ACE2 (<2.50 ng/mL) N = 207	High serum ACE2 (≥2.50 ng/mL) N = 207	*P* value
AKI, 48h after ICU admission	313 (75.6)	144 (69.6)	169 (81.6)	0.006
AKI required RRT	67 (16.2)	23 (11.1)	44 (21.3)	0.007
Length of ICU stay (days)	9.0 (5.0-15.0)	8.0 (5.0-15.0)	9.0 (5.0-14.5)	0.621
Length of hospitalization (days)	25.0 (13.0-42.0)	22.5 (12.3-36.8)	26.0 (14.0-50.0)	0.138
Mortality, in ICU	102 (24.7)	36 (17.5)	66 (31.9)	0.001
Mortality, 90-days	189 (45.7)	82 (39.6)	107 (51.7)	0.018

AKI, acute kidney injury; ICU, intensive care unit; RRT, renal replacement therapy.

### Clinical factors associated with ICU mortality

Univariate analysis revealed significant associations of ICU mortality with high circulating ACE2 concentrations (OR 2.21, 95% CI 1.39–3.51, **p* *= 0.001), the SOFA score (OR 1.41, 95% CI 1.28–1.55, **p* *< 0.001), the mean arterial pressure (OR 0.96, 95% CI 0.94–0.98, **p* *< 0.001), vasopressor/inotrope usage (OR 3.31, 95% CI 2.08–5.27, **p* *< 0.001), fluid resuscitation within 24 hours (OR 1.46, 95% CI 1.28–1.67, **p* *< 0.001), underlying hypertension (OR 0.45, 95% CI 0.29–0.72, **p* *< 0.001), diabetes mellitus (OR 0.48, 95% CI 0.29–0.81, **p* *= 0.005), and the Hb (OR 0.89, 95% CI 0.79–1.00, **p* *= 0.044), TBIL (OR 1.11, 95% CI 1.05–1.17, **p* *< 0.001), and lactate (OR 1.03, 95% CI 1.01–1.04, **p* *< 0.001) concentrations (Table 4). In addition, subgroup analysis showed no significant differences in the association between ACE2 levels and ICU mortality across parents stratified by age or the presence of septic shock (S2 Table). Multivariate regression analysis adjusted for variables with **p* *< 0.05 in the univariate analysis revealed a shift in factors associated with ICU mortality; hypertension and the Hb and TBIL concentrations no longer showed significant associations, whereas high serum ACE2 concentrations did (adjusted OR 1.75, 95% CI 1.00–3.06, **p* *= 0.050). Additionally, the SOFA score (adjusted OR 1.26, 95% CI 1.14–1.40, **p* *< 0.001), mean arterial pressure (adjusted OR 0.97, 95% CI 0.95–1.00, **p* *= 0.025), and fluid resuscitation within the first 24 hours (adjusted OR 1.18, 95% CI 1.01–1.39, **p* *= 0.040) maintained significant associations with ICU mortality (Table 4).

**Table 4:** Univariate and multivariate associations between ICU mortality and clinical variables.

	Univariate		Multivariate*	
	Crude OR (95% CI)	*P*	Adjusted OR (95% CI)	*P*
Serum ACE2				
Low (<2.50 ng/mL)	Ref	Ref	Ref	Ref
High (≥2.50 ng/mL)	2.21 (1.39-3.51)	0.001	1.75 (1.00-3.06)	0.050
Age (years)	0.99 (0.97-1.00)	0.146		
Male gender	0.99 (0.62-1.58)	0.964		
SOFA scores	1.40 (1.28-1.54)	<0.001	1.26 (1.14-1.40)	<0.001
Main arterial pressure (mmHg)	0.96 (0.94-0.98)	<0.001	0.97 (0.95-1.00)	0.025
Vasopressor/inotrope usage	3.31 (2.08-5.27)	<0.001	1.41 (0.79-2.52)	0.243
Fluid resuscitation within 24h (L)	1.46 (1.28-1.67)	<0.001	1.18 (1.01-1.39)	0.040
Invasive mechanical ventilation	1.09 (0.52-2.30)	0.817		
Respiratory tract infection	0.68 (0.40-1.16)	0.159		
Urinary tract infection	0.66 (0.33-1.33)	0.247		
Intra-abdominal infection	1.12 (0.61-2.06)	0.707		
Bloodstream infection	1.18 (0.72-1.94)	0.506		
Hypertension	0.45 (0.29-0.72)	0.001	0.63 (0.36-1.11)	0.112
Diabetic mellitus	0.48 (0.29-0.81)	0.005	0.65 (0.35-1.21)	1.171
Heart failure	0.53 (0.25-1.12)	0.097		
Chronic kidney disease	0.65 (0.39-1.08)	0.096		
Cirrhosis	1.24 (0.53-2.90)	0.623		
Malignancy	1.32 (0.83-2.10)	0.237		
Usage of ACEi/ ARB	0.63 (0.36-1.11)	0.111		
White blood cells (K)	0.99 (0.96-1.02)	0.492		
Hemoglobin (mg/dL)	0.89 (0.79-1.00)	0.046	0.98 (0.85-1.12)	0.727
Creatinine (mg/dL)	0.95 (0.86-1.05)	0.276		
Total bilirubin (mg/dL)	1.11 (1.05-1.17)	<0.001	1.02 (0.97-1.08)	0.462
Lactate (mg/dL)	1.03 (1.02-1.04)	<0.001	1.01 (1.00-1.02)	0.153

*Adjusted for variables with **p* *< 0.05 in the univariate analysis.

ICU, intensive care unit; ACE2, angiotensin converting enzyme 2; SOFA, Sequential Organ Failure Assessment; ACEi, Angiotensin converting enzyme inhibitors; ARB, angiotensin II receptor blockers.

### Clinical factors associated with AKI within 48 hours after ICU admission

Univariate analysis showed that AKI development within 48 hours after ICU admission was associated significantly with high serum ACE2 concentrations (OR 1.95, 95% CI 1.23–3.08, **p* *= 0.005), male sex (OR 0.42, 95% CI 0.25–0.72, **p* *= 0.001), the SOFA score (OR 1.33, 95% CI 1.22–1.46, **p* *< 0.001), UTI (OR 5.28, 95% CI 1.87–14.96, **p* *= 0.002), bloodstream infection (OR 1.98, 95% CI 1.13–3.48, **p* *= 0.018), underlying diabetes mellitus (OR 2.17, 95% CI 1.29–3.64, **p* *= 0.003), pre-existing chronic kidney disease (OR 1.86, 95% CI 1.11–3.13, **p* *= 0.019), and the Hb (OR 0.77, 95% CI 0.70–0.86, **p* *< 0.001), serum Cr (OR 2.36, 95% CI 1.78–3.11, **p* *< 0.001), TBIL (OR 1.11, 95% CI 1.00–1.24, **p* *= 0.049), and lactate (OR 1.05, 95% CI 1.02–1.08, **p* *= 0.001) concentrations (Table 5). No significant association was observed with the patient age, mean arterial pressure, vasopressor/inotrope usage, fluid resuscitation within 24 hours, mechanical ventilation, RTI, IAI, hypertension, heart failure, liver cirrhosis, active malignancy, or WBC. Subgroup analysis revealed that the association between ACE2 levels and the occurrence of AKI did not significantly differ across patient stratified by age or the presence of septic shock (S3 Table). In the adjusted multivariate analysis, elevated serum ACE2 concentrations, the proportion of bloodstream infections, pre-existing chronic kidney disease, and Hb and TBIL concentrations were no longer associated independently AKI development. Factors associated significantly with AKI development within 48 hours after ICU admission were male sex (OR 0.52, 95% CI 0.28–0.96, **p* *= 0.036), the SOFA score (OR 1.23, 95% CI 1.10–1.38, **p* *< 0.001), the proportion of UTIs (OR 3.72, 95% CI 1.19–11.63, **p* *= 0.024), underlying diabetes mellitus (OR 1.89, 95% CI 1.01–3.56, **p* *= 0.047), the serum Cr concentration (OR 2.17, 95% CI 1.52–3.08, **p* *< 0.001), and the lactate concentration (OR 1.04, 95% CI 1.01–1.08, **p* *= 0.008; Table 5).

**Table 5:** Univariate and multivariate associations between acute kidney injury within 48 hours after ICU admission and various clinical variables among critically ill patients.

	Univariate		Multivariate*	
	Crude OR (95% CI)	*P*	Adjusted OR (95% CI)	*P*
Serum ACE2				
Low (<2.50 ng/mL)	Ref	Ref	Ref	Ref
High (≥2.50 ng/mL)	1.95 (1.23-3.08)	0.005	1.17 (0.66-2.09)	0.592
Age (years)	1.01 (0.99-1.02)	0.548		
Male gender	0.42 (0.25-0.72)	0.001	0.52 (0.28-0.96)	0.036
SOFA scores	1.33 (1.22-1.46)	<0.001	1.23 (1.10-1.38)	<0.001
Main arterial pressure (mmHg)	0.99 (0.97-1.00)	0.111		
Vasopressor/inotrope usage	1.27 (0.80-2.01)	0.321		
Fluid resuscitation within 24h (L)	1.13 (0.98-1.30)	0.098		
Invasive mechanical ventilation	0.46 (0.19-1.12)	0.085		
Respiratory tract infection	0.54 (0.29-1.00)	0.052		
Urinary tract infection	5.28 (1.87-14.96)	0.002	3.72 (1.19-11.63)	0.024
Intra-abdominal infection	1.18 (0.62-2.24)	0.610		
Bloodstream infection	1.98 (1.13-3.48)	0.018	0.91 (0.46-1.80)	0.779
Hypertension	1.41 (0.90-2.21)	0.138		
Diabetic mellitus	2.17 (1.29-3.64)	0.003	1.89 (1.01-3.56)	0.047
Heart failure	2.16 (0.99-4.73)	0.054		
Chronic kidney disease	1.86 (1.11-3.13)	0.019	0.62 (0.30-1.29)	0.201
Cirrhosis	2.01 (0.68-5.95)	0.205		
Malignancy	0.75 (0.47-1.19)	0.219		
Usage of ACEi/ ARB	1.13 (0.66-1.94)	0.653		
White blood cells (K)	1.01 (0.98-1.04)	0.420		
Hemoglobin (mg/dL)	0.77 (0.70-0.86)	<0.001	0.89 (0.78-1.01)	0.071
Creatinine (mg/dL)	2.36 (1.78-3.11)	<0.001	2.17 (1.52-3.08)	<0.001
Total bilirubin (mg/dL)	1.11 (1.00-1.24)	0.049	1.02 (0.93-1.13)	0.655
Lactate (mg/dL)	1.05 (1.02-1.08)	0.001	1.04 (1.01-1.08)	0.008

*Adjusted for variables with **p* *< 0.05 in the univariate analysis.

ICU, intensive care unit; ACE2, angiotensin converting enzyme 2; SOFA, Sequential Organ Failure Assessment; ACEi, Angiotensin converting enzyme inhibitors; ARB, angiotensin II receptor blockers.

### Relationships between circulating ACE2 concentrations, ICU mortality, and AKI

Sensitivity analyses using different ACE2 classification yielded similar findings. Even when patients were grouped into tertiles based on their circulating ACE2 concentrations, septic patients in the highest ACE2 tertile remained significantly associated with higher ICU mortality (S2 Table) and AKI within 48 hours of ICU admission (S3 Table) in the univariate regression analysis. After adjusting for other variables with *p* < 0.05 in the univariate analysis, patients in the highest ACE2 tertile remained independently associated with ICU mortality (adjusted OR 2.07, 95% CI 1.02–4.20, *p* = 0.044). However, patients in the highest ACE2 tertile were no longer associated with the development of AKI in the multivariate analysis (adjusted OR 1.18, 95% CI 0.55–2.52, *p* = 0.672).

**Fig 3:**
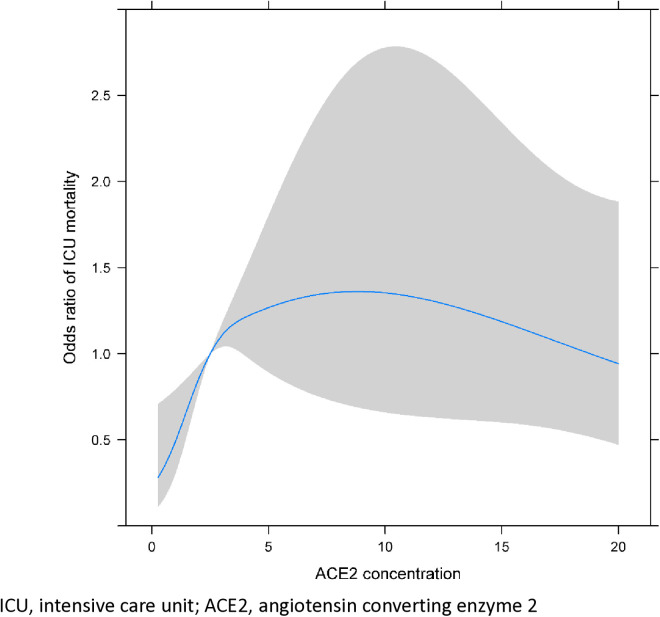
Cubic spline analysis showing the change in odds ratio for ICU mortality in critically ill patients with increased serum ACE2 concentrations (ng/mL).

**Fig 4:**
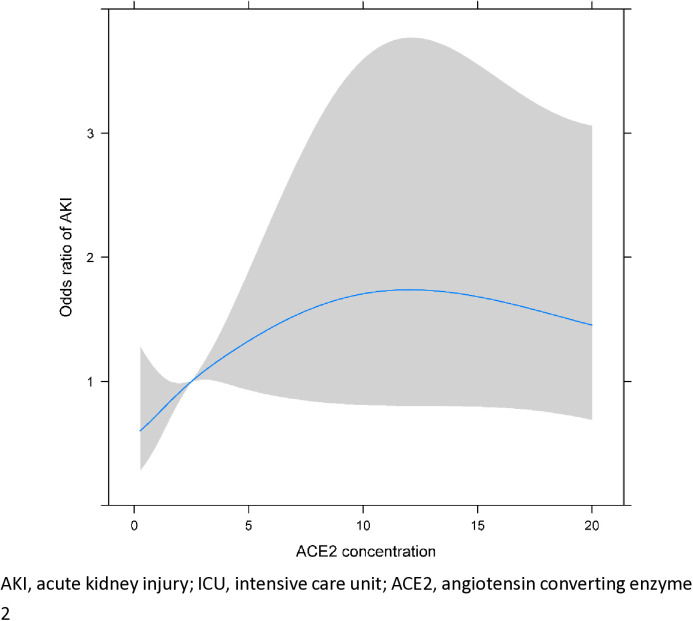
Cubic spline analysis showing the change in odds ratio for AKI within 48 hours after ICU admission in critically ill patients with increased serum ACE2 concentrations (ng/mL).

On the restricted cubic spline plots demonstrating the associations of the ACE2 concentration with ICU mortality and AKI within 48 hours after ICU admission, knots were placed at the serum ACE2 concentrations of 0.2, 1.1, 2.5, 6.6, and 20.0 ng/mL, corresponding to the 2nd, 25th, 50th, 75th, and 98th percentiles. The OR for ICU mortality peaked at the ACE2 concentration of 8.8 ng/mL (OR 1.36, 95% CI 0.69–2.69; Fig 3), and that for AKI peaked at the ACE2 concentration of 12.1 ng/mL (OR 1.74, 95% CI 0.80–3.77; Fig 4).

## Discussion

In contrast to the classic RAS, non-classic RAS pathway functions as a negative regulator of the RAS, with ACE2 playing a pivotal role in this regulation via the degradation of Ang II [1]. Nevertheless, the role of the circulating ACE2 concentrations in single-organ disease, such as renal injury, as well as the etiological or consequential nature of systemic RAS upregulation in the development of AKI, remains not fully understood [1,16,17]. Although the origin of circulating ACE2 remains unclear, researchers have proposed that ACE2 is actively shed from the vascular surface by metalloproteases such as a disintegrin and metalloproteinase (ADAM) 10 and ADAM17, which have been shown to release the enzyme from renal and pulmonary cells in vitro [18], which may reflect the activity of the non-classical RAS pathway in these organs. The final results of this study indicate that patients with confirmed sepsis and circulating ACE2 concentrations ≥ 2.5 ng/mL during the early phase of critical illness have an increased risk of developing AKI within 48 hours of ICU admission and face higher ICU mortality.

ACE2 plays crucial local and systemic roles in renal dysfunction during critical illness. In line with kidney injury, our results showed a 12% increased risk of AKI in patients with higher serum ACE2 levels, along with a 10.1% greater need for renal replacement therapy. Locally, ACE2 normally present in tubular, glomerular, and vascular tissues, shows neo-expression in the glomerular and peritubular capillary endothelium during renal disease or transplantation [16]. On the other hand, increased levels of angiotensinogen and Ang II have been observed in acute tubular necrosis, with the intrarenal RAS correlating with the severity of AKI [19,20]. Given the higher affinity between ACE and Ang I, the conversion of Ang II to Angiotensin-(1–7) is a more common pathway, which consumes ACE2 to reduce elevated Ang II levels and maintain homeostasis [21]. In comparison to the conversion of Ang I to Ang-(1–9), this mechanism leads to a greater depletion of ACE2, and thus serum ACE2 levels may reflect this phenomenon, similar to our results. Evidence has shown that ACE2 mRNA expression in the glomeruli and proximal tubules is reduced, while ACE mRNA expression increases, contributing to the progression of renal injury [17]. These findings underscore ACE2’s role as a critical negative regulator of the intrarenal RAS, helping to maintain homeostasis [22]. Systemically, circulating ACE2 concentrations are found to be reduced in both acute and chronic kidney diseases [1,23], disrupting RAS homeostasis and exacerbating renal damage [24]. It has been suggested that ACE2 and Ang-(1–7) exert renoprotective effects by improving renal blood flow and glomerular filtration rate [25,26]. Previous studies have shown that administering exogenous Ang II to patients with distributive shock and severe AKI is associated with better survival rates and a reduced need for renal replacement therapy, likely due to the reduction in vasodilatory angiotensin levels [3,27]. However, while dysregulation of ACE in sepsis has been discussed, there is limited focus on the downstream products of Ang II and the role of the non-classic RAS cascade.

Early identification of septic patients at risk of AKI is crucial for timely and appropriate intervention. Given the high prevalence of AKI in sepsis and its strong association with poor outcomes, identifying a reliable biomarker for predicting AKI risk following severe sepsis is essential [28]. Both experimental models and human studies have identified several mechanisms in the classic RAS that contribute to these outcomes during septic shock: (1) impaired Ang II production, potentially due to ACE activity deficiencies [29]; (2) increased Ang II degradation by peptidases [30,31]; and (3) reduced AT1 receptor availability caused by internalization, decreased synthesis, or pharmacological blockade [32,33]. One plausible explanation for the association of the ACE2 concentration with outcomes in septic patients is that endothelial injury during shock may lead to ACE deficiencies, resulting in the elevation of vasodilatory mediators typically metabolized by ACE and Ang II [3]. Beyond the classic cascade, ACE2 facilitates the conversion of excess Ang II into Ang-(1–7) in the overactivated RAS [1,34]. Nevertheless, in septic patients with preserved or regained ACE function, ACE maintains the conversion of Ang I to Ang II, suggesting a potential for the reduction of endothelial injury or restoration of endothelial function [35]. These findings suggest that elevated circulating ACE2 concentrations represent a compensatory mechanism, counterbalancing increases in the Ang II concentration observed during severe sepsis when ACE activity is decreased. In studies of RAS cascade balance in sepsis models, ACE2-knockout mice had worse prognoses than did ACE2–knock-in mice [36]. ACE2 on myeloid cells protects against sepsis-induced hypotension and vascular dysfunction following bone marrow transplantation [36]. This protective role also extends to recombinant human ACE2 administered during sepsis-induced cardiac dysfunction [37]. In contrast to the diabetic model, where the aforementioned mechanisms are not present, an increase in serum Ang II concentration has been associated with elevated circulating ACE and ACE2 activity in early diabetes, with a more pronounced increase in ACE2 than in ACE, although this elevation is insufficient to prevent diabetes-related kidney injury due to Ang II overactivity [34].

Our findings suggest that lower serum ACE2 concentrations in septic patients reflect a less-activated RAS and preserved ACE function, as evidenced by a reduced incidence of AKI within 48 hours of ICU admission. Moreover, high ACE2 concentrations at the time of ICU admission appear to correlated with ICU mortality, underscoring the potential clinical significance of ACE2 measurement in critically ill patients with sepsis. This prognostic value may aid the stratification of disease severity in this population and guide the adjustment of RAS-acting agents to improve patient outcomes. Previous studies have shown that Ang II and renin levels in sepsis correlate significantly with disease severity and mortality [38–41]. However, rather than solely monitoring upstream components of RAS, ACE2 levels may serve as a more direct indicator of non-classic cascade activity, which is linked to RAS downregulation. This perspective aligns with our findings, where patients in the low ACE2 group, potentially experiencing greater ACE2 depletion, exhibited higher survival rates.

In this study, the risk of AKI was greater in the high ACE2 group but the ACE2 concentration was not an independent risk factor after adjustment for confounding variables, possibly due to the influence of the SOFA score and serum Cr concentration, which are comprehensive measures of organ function and kidney health. The SOFA score, which integrates multiple physiological factors, may overshadow ACE2’s isolated impact, and the serum Cr level, closely linked to kidney function, could have reduced ACE2’s apparent effect in the analysis. Thus, whereas the ACE2 concentration correlates with the AKI risk, we believe that AKI is not caused by a single factor but rather a combination of multiple influences. The ACE2 level should be considered a predictive marker rather than a direct causative factor for AKI.

Since ACE2 is widely recognized for its tissue-protective functions, it also serves as the primary entry receptor for coronaviruses, particularly SARS-CoV-2, through its spike glycoprotein, facilitating viral entry into host cells in the lungs [21,42] Subsequent to infection, ACE2 expression on host cells is downregulated, leading to an imbalance between the classical and non-classical RAS pathways, which contributes to multi-organ dysfunction. Infected cells, along with immune cells activated by viral antigens, secrete pro-inflammatory cytokines and chemokines, thereby initiating immune and inflammatory responses aimed at combating the virus [42]. Furthermore, both free and macrophage-phagocytosed viral particles in the bloodstream can disseminate to other organs, where they may infect ACE2-expressing cells at local sites [42]. The inclusion process for this study began during the experimental design phase and concluded before the local COVID-19 outbreak, minimizing the potential influence of SARS-CoV-2. However, other occult viral infections cannot be ruled out, especially given that respiratory tract infections are the leading cause of sepsis.

Certain limitations of this study warrant acknowledgment. Our cohort consisted mainly of older, more severely ill patients, which may introduce confounding due to factors like chronic kidney disease and diabetes, both linked to higher serum ACE2 levels [43–46]. Although ACEi/ARB and vasopressor/inotrope use showed no significant effect, residual confounding from unmeasured factors, such as prior medication use, fluid resuscitation, and organ function changes, cannot be ruled out. Second, no circulating ACE2 concentration threshold for predicting outcomes in septic populations has been established. The cutoff value of 2.5 ng/mL used in this study was based on the median serum ACE2 concentration of all enrolled subjects. We employed a cubic spline approach to examine the continuous relationship between circulating ACE2 concentrations and clinical outcomes. Third, the serum ACE2 concentration may not directly reflect the effect of ACE2 in the kidney or RAS. In addition, the serum ACE2 concentration does not necessarily correlate with circulating ACE2 activity due to the potential presence of circulating inhibitors. Further studies are needed to investigate circulating RAS peptides and the possible influence of serum ACE2 on specific organs. Finally, causality cannot be established in this observational study, and the generalizability of our findings remains uncertain without external validation. Prospective interventional trials are also needed to clarify the relationship between circulating ACE2 and critical illness.

## Conclusions

The discernment of ACE2’s utility as a prognostic biomarker in a septic population in this study provides important insight and suggests that the activation of the non-classic RAS has predictive value. The exploration of the dynamic interplay occurring within the RAS, especially the delicate balance between its classic and non-classic components, may open avenues for therapeutic interventions and further our understanding of the pathophysiology of critical illnesses.

## Supporting information

S1 Table Numbers of study subjects with missing data before imputation. Subjects with missing data are expressed as numbers (percentage).
(DOCX)

S2 Table Subgroup analysis to investigate the association between high ACE2 levels and ICU mortality in critically ill patients stratified by different age and severity of sepsis. (DOCX)

S3 Table Subgroup analysis to investigate the association between high ACE2 levels and acute kidney injury in critically ill patients stratified by different age and severity of sepsis. (DOCX)
