# Supplementary material for: Circulating angiotensin-converting enzyme 2 concentration is associated with acute kidney injury and mortality in sepsis
Source: PLoS One. 2025 Aug 29;20(8):e0330668. doi: 10.1371/journal.pone.0330668 (PMC12396652; doi:10.1371/journal.pone.0330668)
Supplement: S1 Table — Subjects with missing data are expressed as numbers (percentage). (DOCX) [file pone.0330668.s001.docx]

**Supporting Table 1.** Numbers of study subjects with missing data before imputation. Subjects with missing data are expressed as numbers (percentage).

|  | **Total population**  N=414 | **Mean of non-missing subjects before imputation** |
| --- | --- | --- |
| Age (years) | 0 | 68.3 |
| Male gender | 0 |  |
| SOFA scores | 84 (20.3) | 9.5 |
| Invasive mechanical ventilation | 0 |  |
| Main arterial pressure (mmHg) | 0 | 56.7 |
| Vasopressor/inotrope usage | 0 |  |
| Fluid resuscitation within 24h (L) | 0 | 2.2 |
| **Sources of Infection** |  |  |
| Respiratory tract infection | 0 |  |
| Urinary tract infection | 0 |  |
| Intra-abdominal infection | 0 |  |
| Bloodstream infection | 0 |  |
| **Co-morbidities** |  |  |
| Hypertension | 0 |  |
| Diabetic mellitus | 0 |  |
| Heart failure | 0 |  |
| Chronic kidney disease | 0 |  |
| Cirrhosis | 0 |  |
| Malignancy | 0 |  |
| **Lab data** |  |  |
| White blood cells (K) | 8 (1.9) | 10.6 |
| Hemoglobin (mg/dL) | 6 (1.4) | 9.2 |
| Creatinine (mg/dL) | 42 (10.1) | 2.6 |
| Total bilirubin (md/dL) | 33 (8.0) | 2.2 |
| Lactate (mg/dL) | 63 (15.2) | 18.9 |
| ACE2 (ng/mL) | 0 | 5.4 |

SOFA, Sequential Organ Failure Assessment.
