# Supplementary material for: Circulating angiotensin-converting enzyme 2 concentration is associated with acute kidney injury and mortality in sepsis
Source: PLoS One. 2025 Aug 29;20(8):e0330668. doi: 10.1371/journal.pone.0330668 (PMC12396652; doi:10.1371/journal.pone.0330668)
Supplement: S2 Table — (DOCX) [file pone.0330668.s002.docx]

|  | **High (≥2.50 ng/mL) *vs*. low (<2.50 ng/mL) ACE2** | | | |
| --- | --- | --- | --- | --- |
| **Subgroup (event/subjects, %)** | **Crude OR** | **95% CI** | ***P* effect** | ***P* interaction** |
| **Overall** (102/414, 24.6%) | 2.21 | 1.39-3.51 | 0.001 |  |
| **Age ≥ 65 years old** |  |  |  |  |
| No (47/162, 29.0%) | 1.99 | 0.99-3.99 | 0.052 | 0.668 |
| Yes (55/252, 21.8%) | 2.44 | 1.30-4.59 | 0.005 |  |
| **With septic shock** |  |  |  |  |
| No (63/329, 19.1%) | 1.99 | 1.14-3.50 | 0.016 | 0.699 |
| Yes (39/85, 45.9%) | 2.46 | 1.01-5.99 | 0.049 |  |

**Supporting Table 2.** Subgroup analysis to investigate the association between high ACE2 levels and ICU mortality in critically ill patients stratified by different age and severity of sepsis.

ACE2, angiotensin-converting enzyme 2; ICU, intensive care units; OR, Odds ratio; CI, confidence interval
