# Supplementary material for: Circulating angiotensin-converting enzyme 2 concentration is associated with acute kidney injury and mortality in sepsis
Source: PLoS One. 2025 Aug 29;20(8):e0330668. doi: 10.1371/journal.pone.0330668 (PMC12396652; doi:10.1371/journal.pone.0330668)
Supplement: S3 Table — (DOCX) [file pone.0330668.s003.docx]

|  | **High (≥2.50 ng/mL) *vs*. low (<2.50 ng/mL) ACE2** | | | |
| --- | --- | --- | --- | --- |
| **Subgroup (event/subjects, %)** | **Crude OR** | **95% CI** | ***P* effect** | ***P* interaction** |
| **Overall** (313/414, 75.6%) | 1.95 | 1.23-3.08 | 0.005 |  |
| **Age ≥ 65 years old** |  |  |  |  |
| No (120/162, 74.1%) | 1.86 | 0.91-3.81 | 0.091 | 0.871 |
| Yes (193/252, 76.6%) | 2.01 | 1.10-3.65 | 0.023 |  |
| **With septic shock** |  |  |  |  |
| No (235/329, 71.4%) | 1.85 | 1.13-3.02 | 0.014 | 0.967 |
| Yes (78/85, 91.8%) | 1.92 | 0.40-9.15 | 0.415 |  |

**Supporting Table 3.** Subgroup analysis to investigate the association between high ACE2 levels and acute kidney injury in critically ill patients stratified by different age and severity of sepsis.

ACE2, angiotensin-converting enzyme 2; OR, Odds ratio; CI, confidence interval
